# Weight change and mortality and cardiovascular outcomes in patients with new-onset diabetes mellitus: a nationwide cohort study

**DOI:** 10.1186/s12933-019-0838-9

**Published:** 2019-03-19

**Authors:** Mee Kyoung Kim, Kyungdo Han, Eun Sil Koh, Eun Sook Kim, Min-Kyung Lee, Ga Eun Nam, Hyuk-Sang Kwon

**Affiliations:** 1 Division of Endocrinology and Metabolism, Department of Internal Medicine, Yeouido St. Mary’s Hospital, College of Medicine, The Catholic University of Korea, #10 63-ro, Yeongdeungpo-gu, Seoul, 07345 South Korea; 2 Department of Medical Statistics, College of Medicine, The Catholic University of Korea, Seoul, 06591 South Korea; 3 Division of Nephrology, Department of Internal Medicine, Yeouido St. Mary’s Hospital, College of Medicine, The Catholic University of Korea, Seoul, 07345 South Korea; 4 Division of Endocrinology and Metabolism, Department of Internal Medicine, Incheon St. Mary’s Hospital, College of Medicine, The Catholic University of Korea, Incheon, 21431 South Korea; 5 Division of Endocrinology and Metabolism, Department of Internal Medicine, Myongji Hospital, Goyang-Si, Gyeonggi-do 10475 South Korea; 6 Department of Family Medicine, Sahmyook Medical Center, Seoul, 02500 South Korea

**Keywords:** Weight changes, Type 2 diabetes mellitus, Cardiovascular disease

## Abstract

**Background:**

Because weight control is a cornerstone of diabetes management, it is important to understand the relationship of weight change to risk of cardiovascular disease (CVD) among patients with type 2 diabetes mellitus (DM). We aimed to investigate whether changes in weight early after diagnosis influence the incidence of CVD and all-cause mortality in patients with type 2 DM.

**Methods:**

Using nationally representative data from the Korean National Health Insurance System, 173,246 subjects with new-onset DM who underwent health examinations during 2007–2012 were included. Weight was measured at the time of diabetes diagnosis and 2 years later. Weight change over 2 years was divided into five categories of 5% weight change, from weight loss ≥ − 10% to weight gain ≥ 10%.

**Results:**

There were 3113 deaths (1.8%), 2060 cases of stroke (1.2%), and 1767 myocardial infarctions (MIs) (1.0%) during a median follow-up of 5.5 years. Subjects with weight gain ≥ 10% had a significantly higher risk of stroke (hazard ratio [HR] 1.51, 95% confidence interval [CI] 1.23–1.84), compared with the group with stable weight. There was no significant association between weight change after diagnosis of DM and incident MI. All-cause mortality showed a U-shaped curve according to weight change. The group with weight loss ≥ − 10% had the highest HR for all-cause mortality (HR 1.86; 95% CI 1.61–2.14) and the HR for weight gain ≥ 10% was 1.61 (95% CI 1.37–1.89).

**Conclusions:**

Weight changes of more than 10% after diabetes diagnosis were associated with higher mortality and over 10% weight gain was associated with increased risk of stroke.

**Electronic supplementary material:**

The online version of this article (10.1186/s12933-019-0838-9) contains supplementary material, which is available to authorized users.

## Introduction

Bariatric surgery for severe obesity in patients with type 2 diabetes mellitus (DM) that leads to substantial weight loss over several years has been shown to reduce significantly the risk of diabetic complications and mortality compared with untreated patients [1]. However, the large randomized Look Action for Health in Diabetes (AHEAD) trial was unable to confirm the health benefits related to weight loss [2]. Results from Look AHEAD showed that despite a greater weight loss with diet and exercise in the intervention group compared with the control group, neither mortality nor cardiovascular morbidity was reduced [2]. However, the clinical effect of weight changes on cardiovascular disease (CVD) or mortality in the context of routine lifestyle changes in general diabetes care has previously been debated and remains unclear [3–5].

Because obesity is highly prevalent among people with type 2 DM and weight control is a cornerstone of diabetes management, it is important to understand the relationship of weight change to the risk of CVD among patients with type 2 DM [6]. In the Cardiovascular Health Study, the diagnosis of diabetes among individuals aged 65 years and older was associated with a hazard ratio (HR) of 2.3 for mortality within the first 2 years after the diagnosis [7, 8]. The initial period after a diagnosis of type 2 DM may be especially critical for early glycemic control. Therefore, in the present study we aimed to investigate the association between weight change during the first 2 years after diagnosis of DM on all-cause mortality, myocardial infarction (MI), and stroke.

## Materials and methods

### Data source and study population

The Korean NHIS comprises a complete set of health information pertaining to about 50 million Koreans. The NHIS includes an eligibility database (age, sex, socioeconomic variables, type of eligibility, etc.); a medical treatment database (based on the accounts submitted by medical service providers for medical expenses); a health examination database (results of general health examinations and questionnaires on lifestyle and behavior); a medical care institution database (types of medical care institutions, location, equipment, and number of physicians); and death information [9–12]. The dates of death were obtained from the eligibility database, which was prepared by Statistics Korea [13]. In Korea, the NHIS is the single insurer, is managed by the government, and covers all Koreans. The NHIS is consisted of employee subscribers and regional insurance subscribers. All examinees were requested to have biannual health checkups, but employee subscribers are requested to have annual examination [11].

**Fig. 1:**
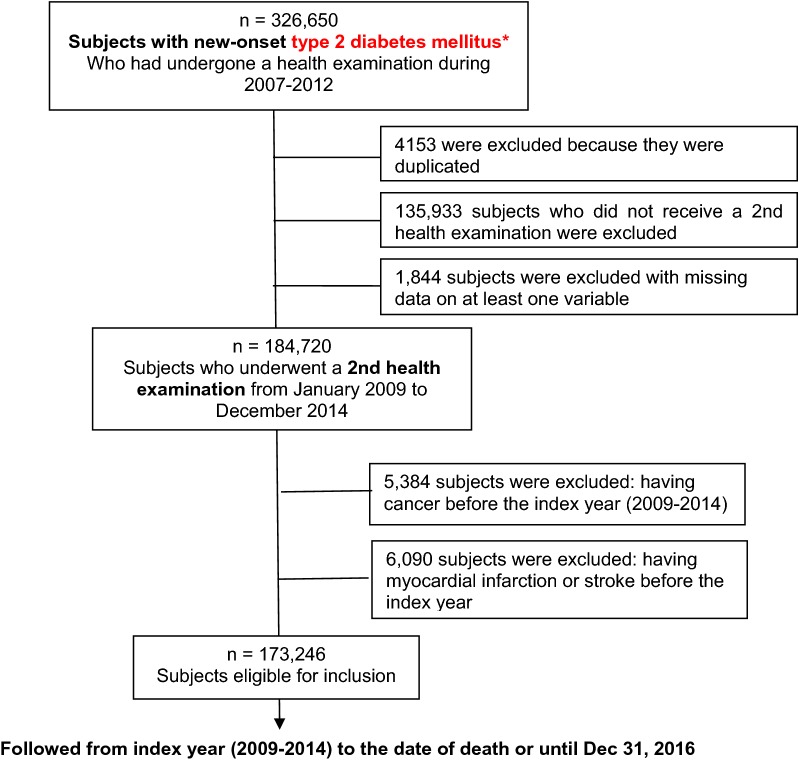
Flow chart of the study population. * New-onset type 2 diabetes mellitus was defined as follows: (1) had never previously taken any diabetic medications; (2) had an fasting blood glucose (FBG) ≥ 126 mg/dL at a health examination during 2007–2012; (3) started taking diabetic medications within 1 year of their health examination under the International Classification of Diseases, 10th Revision (ICD-10) codes E11–14. Patients with the disease code insulin-dependent DM (E10) was excluded

In our study, we included subjects (aged ≥ 30 years) who had undergone a health examination between 2007 and 2012. Among these, we included only subjects with new-onset type 2 DM who started taking diabetic medications within 1 year of their health checkup. We defined new-onset type 2 DM as follows: (1) had never previously taken any diabetic medications; (2) had an fasting blood glucose (FBG) ≥ 126 mg/dL at a health examination during 2007–2012; (3) started taking diabetic medications within 1 year of their health examination under the International Classification of Diseases, 10th Revision (ICD-10) codes E11–14. Patients were classified as having type 2 DM when they had at least one service claim with a diagnosis of type 2 DM based on ICD-10 codes E11, E12, E13, or E14, as either the principal diagnosis or the first to fourth additional diagnosis, and were prescribed at least one antidiabetic drug anytime in a given year to exclude prediabetes or non-diabetic subjects. We excluded patients with the disease code insulin-dependent DM (E10). To validate the information accuracy, an expert committee from the Korean Diabetes Association reviewed the dataset regularly. The committee decided the suitability of the dataset [14]. Of 326,650 subjects with new-onset DM, 184,720 underwent a second health examination during 2009–2014 (index year). To avoid confounding the data by effects of preexisting diseases and to minimize the possible effects of reverse causality, individuals who had a history of MI (ICD–10 codes: I21, I22) or stroke (ICD–10 codes: I63, I64) before the index year were also excluded (n = 6090). The presence of malignancy may contribute to weight change so patients with any malignancy (n = 5384) were also excluded. Ultimately, the study population consisted of 173,246 subjects (Fig. 1). This study was approved by the Institutional Review Board of The Catholic University of Korea (No. SC18ZES10009). Anonymous and deidentified information was used for analysis and, therefore, informed consent was not obtained.

### Measurements and definitions

The general medical examination included history taking, blood pressure (BP) measurement, blood sampling, urinalysis, and chest X-ray. According to the protocol, BP was measured by a trained clinician after the participant had been seated for 5 min with an arm in the appropriate position. Blood samples for the measurement of serum glucose and lipid levels were drawn after an overnight fast. The hospitals in which these health examinations were performed were certified by the NHIS and subjected to regular quality control. Hypertension was defined as BP ≥ 140/90 mmHg or at least one claim per year for an antihypertensive medication prescription under ICD-10 codes I10–I15. Dyslipidemia was defined as total cholesterol level ≥ 240 mg/dL or at least one claim per year for an antihyperlipidemic medication prescription under ICD-10 code E78. Information on current smoking and alcohol consumption (heavy alcohol consumption defined as ≥ 30 g/day) was obtained by questionnaire. Regular exercise was defined as performing > 30 min of moderate physical activity at least five times per week or > 20 min of strenuous physical activity at least three times per week. Income level was dichotomized at the lowest 25%.

### Categories of weight change

Weight change was calculated as the difference in body weight over 2 years, corresponding to the period between the first and second health checkup. For example, weight change over 2 years was checked for 2007 through 2009, 2008 through 2010, 2009 through 2011, 2010 through 2012, 2011 through 2013, and 2012 through 2014. Based on the study performed by Corrada et al. [15] and Kim et al. [16] we defined the weight-stable group as those with < 5% weight change, and we categorized weight change into five weight change groups by 5% increase or decrease as follows: weight loss ≥ 10%; loss ≥ 5% to < 10%; weight change < 5%; gain ≥ 5% to < 10%; gain ≥ 10%. The body mass index (BMI) was calculated as weight in kilograms divided by the square of the height in meters. Individuals were categorized into five BMI groups: < 18.5 kg/m^2^ (underweight), 18.5–22.9 kg/m^2^ (normal), 23.0–24.9 kg/m^2^ (overweight), 25.0–29.9 kg/m^2^ (class I obese), and ≥ 30 kg/m^2^ (class II obese), according to the World Health Organization recommendations for Asians [17, 18].

### Study outcomes and follow-up

The end points of the study were newly diagnosed MI, stroke, or death. MI was defined as the hospitalization with myocardial infarction diagnostic codes of I21–22. Stroke was defined as the recording of ICD-10 codes I63 or I64 during hospitalization with claims for brain magnetic resonance imaging or brain computed tomography. Subjects without MI or stroke during their follow-up periods were considered to have completed the study at the date of their death or at the end of follow-up, whichever came first. If an MI or stroke event occurred before death, it was included in MI or stroke cases. The study population was followed from baseline to the date of death or cardiovascular events, or until December 31, 2016, whichever came first.

### Statistical analysis

Baseline characteristics are presented as the mean ± SD or n (%). Participants were classified into five weight-change categories. The incidence rate of primary outcomes was calculated by dividing the number of incident cases by the total follow-up duration (person-years). The survival and disease-free probability of primary outcomes according to the weight-change categories was calculated using Kaplan–Meier curves, and a log-rank test was performed to analyze differences among the groups. Hazard ratios (HRs) and 95% confidence interval (CI) values for all-cause mortality, MI, and stroke were calculated using a Cox proportional hazards model for weight-change categories or decile groups of weight change. The proportional-hazards assumption was evaluated using the Schoenfeld residuals test with the logarithm of the cumulative hazards function based on Kaplan–Meier estimates for weight-change categories. There was no significant departure from proportionality in hazards over time. A multivariable-adjusted proportional hazards model was applied. Model 1 was adjusted for age and sex; Model 2 was adjusted further for smoking, alcohol consumption, regular exercise, and income status; and Model 3 was adjusted further for baseline FBG, dyslipidemia, hypertension, waist circumference, baseline use of insulin, and estimated glomerular filtration rate. We performed competing risk survival analysis including mortality as a competing risk (calculating survival probability using sub-distribution hazards model [Fine and Gray method]). A sensitivity analysis was performed to exclude participants with end points occurring within 1 year of follow-up to account for the possibility of reverse causation. The potential effect modification by age, sex, obesity, and smoking was evaluated using stratified analysis and interaction testing using a likelihood-ratio test. Restricted cubic spline curves were used to visualize the relationship between body weight change and the probability of outcomes occurrence with the use of the ‘Hmisc’ packages in R program. Statistical analyses were performed using SAS version 9.4 (SAS Institute Inc., Cary, NC, USA), and a P value < 0.05 was considered to indicate significance.

## Results

### Baseline characteristics of the study population

The characteristics of the participants grouped according to their weight change during the 2 years after diagnosis of DM are listed in Table 1.

**Table 1:** Baseline characteristics of subjects according to the weight-changes for 2 years after diagnosis of diabetes mellitus

Weight change (%)	≥ − 10%	− 10 ~ − 5%	− 5 ~ 5%	5 ~ 10%	≥ 10%
	(n = 6897)	(n = 25,468)	(n = 118,691)	(n = 16,127)	(n = 6053)
At diagnosis of diabetes mellitus					
Height (cm)	161.6 ± 9.6	162.7 ± 9.3	164.1 ± 8.8	164.5 ± 8.8	164.6 ± 8.8
Body weight (kg)	69.6 ± 13.1	69.3 ± 12.1	69.5 ± 11.4	66.7 ± 11.4	63.3 ± 11.1
FBG (mg/dL)	163.5 ± 41.3	162.6 ± 40.0	170.1 ± 45.5	202.5 ± 59.9	230.7 ± 66.7
Systolic BP (mmHg)	130.4 ± 16.2	130.0 ± 15.5	130.0 ± 15.6	129.2 ± 16.1	127.5 ± 16.5
Diastolic BP (mmHg)	80.6 ± 10.4	80.7 ± 10.2	81 ± 10.2	80.7 ± 10.5	80.2 ± 10.4
Hypertension (yes, %)	3478 (50.5)	12,592 (49.5)	60,598 (51.2)	7632 (47.4)	2561 (42.4)
Total cholesterol (mg/dL)	216.7 ± 42.39	216.19 ± 41.47	215.54 ± 41.8	216.98 ± 43.88	218 ± 46.09
Dyslipidemia (yes)	3229 (46.93)	11,530 (45.34)	52,940 (44.71)	6974 (43.38)	2642 (43.88)
After the first 2 years					
Age (years)	57.2 ± 12.1	56.7 ± 10.9	56.0 ± 10.2	54.9 ± 10.3	54.2 ± 10.8
Sex (male)	3340 (48.4)	14,577 (57.2)	79,477 (67.0)	11,418 (70.8)	4400 (72.7)
BMI (kg/m^2^)	23.01 ± 3.14	24.32 ± 3.04	25.64 ± 3.16	26.25 ± 3.38	26.54 ± 3.57
Height (cm)	161.2 ± 9.7	162.5 ± 9.3	164.0 ± 8.8	164.5 ± 8.8	164.7 ± 8.9
Body weight (kg)	60.05 ± 11.34	64.46 ± 11.27	69.16 ± 11.38	71.25 ± 12.16	72.23 ± 12.49
FBG (mg/dL)	127.6 ± 49	131.3 ± 42.2	137.2 ± 38.5	139.9 ± 38.7	140.6 ± 42.2
Systolic BP (mmHg)	123.6 ± 14.9	125.3 ± 14.4	127.7 ± 14.3	128.9 ± 14.4	129.5 ± 14.9
Diastolic BP (mmHg)	76.3 ± 9.8	77.5 ± 9.5	79.0 ± 9.5	79.8 ± 9.5	80.1 ± 9.6
Hypertension (yes, %)	3267 (47.4)	12,534 (49.3)	64,971 (54.8)	9265 (57.5)	3464 (57.3)
Total cholesterol (mg/dL)	184.0 ± 40.9	188.8 ± 40.7	191.1 ± 40.11	188.5 ± 39.23	188.0 ± 39.6
Dyslipidemia (yes, %)	3036 (44.1)	11,956 (47.0)	59,955 (50.6)	8320 (51.7)	3152 (52.1)
Waist circumferences (cm)	80.4 ± 8.6	83.1 ± 8.1	86.5 ± 8.1	87. 9 ± 8.4	88.5 ± 8.7
Current smoker	1464 (21.3)	6200 (24.4)	32,347 (27.3)	4716 (29.3)	1825 (30.2)
Heavy alcohol drinker	344 (5)	1740 (6.9)	10,871 (9.2)	1596 (9.9)	613 (10.2)
Regular exercise	3652 (53.1)	14,066 (55.3)	67,037 (56.6)	8862 (55.1)	3282 (54.3)
Income (lower 25%)	1485 (21.5)	4962 (19.5)	22,829 (19.2)	3380 (21.0)	1300 (21.5)
Use of insulin (yes)	170 (2.5)	338 (1.3)	1292 (1.1)	345 (2.1)	263 (4.3)

Data are expressed as the mean ± SD, or n (%). P-values for the trend were < 0.0001 for all variables because of the large size of the study population. *BMI* body mass index, *FBG* fasting blood glucose, *BP* blood pressure

During the first 2 years, 68.5% of participants had no change (– 5% to + 5%) in their body weight, while 4.0% decreased their weight by ≥ – 10% and 3.5% increased their weight by ≥ + 10%. In subjects with a BMI ≥ 25 kg/m^2^ at diagnosis of DM, the percent of subjects with weight loss ≥ − 5% was higher than the percent of subjects with weight gain ≥ 5% (Additional file 1: Table S1).

Subjects with weight gain ≥ 10% were younger and more likely to be male, to have higher baseline FBG, systolic BP, and diastolic BP, and to be current smokers and frequent heavy drinkers (Table 1). The highest FBG at the time of diagnosis of DM was observed in participants with weight gain ≥ 10%; in contrast, systolic BP at the diagnosis of DM was not elevated in this group. However, at 2 years after diagnosis of DM, systolic BP was highest in the group with weight gain ≥ 10%.

### Risk of all-cause mortality, MI, and stroke according to the weight change after diagnosis of DM

There were 3113 deaths (1.8%), 2060 cases of stroke (1.2%), and 1767 MIs (1.0%) during a median follow-up of 5.5 years. Group with stable weight (− 5% to + 5%) after diagnosis of DM had the lowest cumulative incidence of all-cause mortality (Additional file 1: Figure S1). All-cause mortality showed a U-shaped curve according to weight change (Table 2). Higher HRs for all-cause mortality were associated with weight loss or weight gain ≥ + 10%. The group with weight loss ≥ − 10% had the highest HR for all-cause mortality (HR, 1.86; 95% CI 1.61–2.14) and the HR for the group with weight gain ≥ + 10% was 1.61 (95% CI 1.37–1.89).

**Table 2:** Hazard ratios and 95% confidence intervals of all-cause mortality, myocardial infarction and stroke by the weight-changes for 2 years after diagnosis of diabetes mellitus

Weight change (%)	Events (n)	Incidence rate (per 1000 person-years)	Model 1	Model 2	Model 3
Myocardial infarction					
≥ − 10%	72	3.05	1.06 (0.84, 1.35)	1.05 (0.83,1.34)	1.08 (0.85, 1.38)
− 10 ~ − 5%	265	3.04	1.06 (0.93, 1.22)	1.07 (0.93,1.22)	1.10 (0.96, 1.27)
− 5 ~ 5%	1188	2.84	1 (ref.)	1 (ref.)	1 (ref.)
5 ~ 10%	174	2.95	1.06 (0.91, 1.25)	1.05 (0.90,1.23)	1.04 (0.88, 1.22)
≥ 10%	68	3.09	1.13 (0.89, 1.45)	1.11 (0.87,1.42)	1.07 (0.84, 1.38)
Stroke					
≥ − 10%	93	3.95	1.07 (0.86, 1.32)	1.05 (0.85,1.30)	1.08 (0.87, 1.34)
− 10 ~ − 5%	278	3.19	0.92 (0.81, 1.05)	0.92 (0.81,1.05)	0.97 (0.85, 1.11)
− 5 ~ 5%	1375	3.29	1 (ref.)	1 (ref.)	1 (ref.)
5 ~ 10%	207	3.51	1.11 (0.96, 1.28)	1.10 (0.95,1.28)	1.08 (0.93, 1.25)
≥ 10%	107	4.89	1.57 (1.29, 1.91)	1.51 (1.23,1.84)	1.47 (1.20, 1.79)
All-cause mortality					
≥ − 10%	224	9.43	2.07 (1.80, 2.38)	1.86 (1.61,2.14)	1.83 (1.59, 2.12)
− 10 ~ − 5%	523	5.96	1.34 (1.22, 1.48)	1.28 (1.16,1.41)	1.27 (1.15, 1.41)
− 5 ~ 5%	1862	4.43	1 (ref.)	1 (ref.)	1 (ref.)
5 ~ 10%	343	5.78	1.30 (1.16, 1.46)	1.27 (1.13,1.43)	1.26 (1.12, 1.41)
≥ 10%	161	7.28	1.65 (1.40, 1.94)	1.61 (1.37,1.89)	1.57 (1.33, 1.85)

Model 1: adjusted for age and sex

Model 2: adjusted for model 1 plus alcohol drinking, smoking, regular exercise and income status

Model 3: adjusted for model 2 plus baseline fasting glucose levels, dyslipidemia, hypertension, waist circumferences, use of insulin and estimated glomerular filtration rate

There was no significant association between weight change after diagnosis of DM and incident MI. Subjects with weight gain ≥ + 10% had a significantly higher risk of stroke (HR 1.51, 95% CI 1.23–1.84) compared with the group with stable weight. The associations between weight gain and outcomes were confirmed after adjusting for baseline FBG, dyslipidemia, hypertension, waist circumference, insulin use and estimated glomerular filtration rate (Table 2; model 3). Because an event of mortality could compete with cardiovascular outcome of interest, we also performed competing risk analysis using a sub-distribution hazards model. Competing risk analysis including mortality as a competing risk showed similar results (Additional file 1: Table S2).

The relationship between body weight change and the risk of MI, stroke, and all-cause mortality was not linear as shown in Additional file 1: Figure S2. Both weight gain or loss were significantly associated with increased risk of all-cause mortality.

The risk of all-cause mortality, MI and stroke increased among individuals with underweight (Additional file 1: Table S3) and the risk of all-cause mortality decreased as BMI increases from 23 to 25 kg/m^2^, 25–30 kg/m^2^, and to ≥ 30 kg/m^2^ compared to group with BMI of 18.5–23 kg/m^2^.

### Sensitivity analysis

**Fig. 2:**
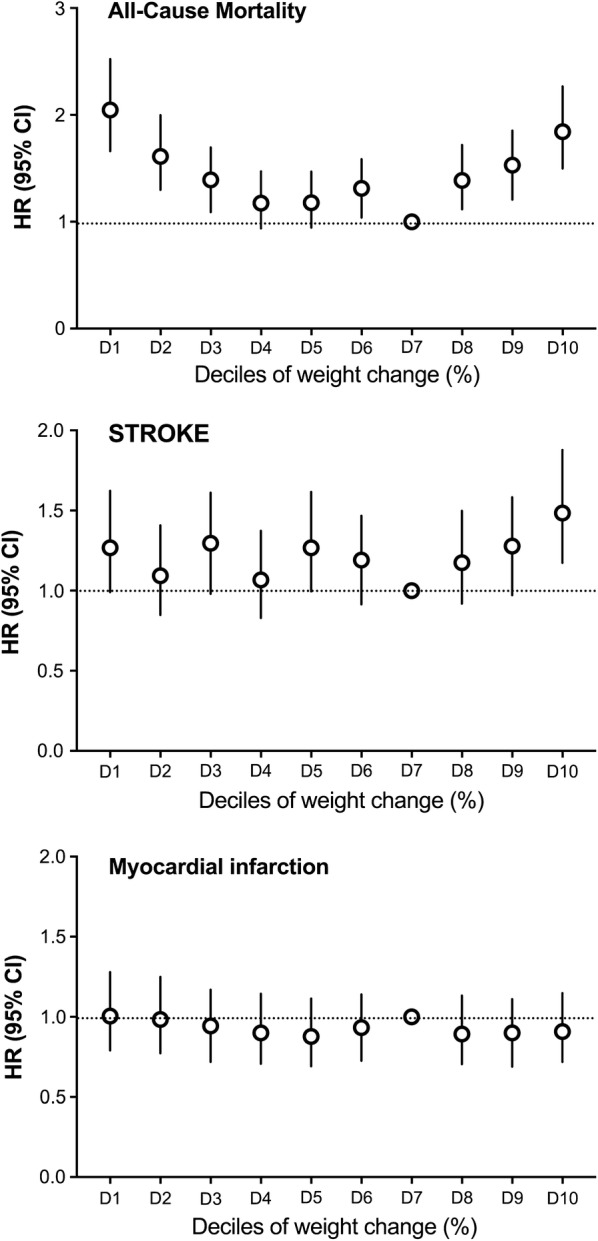
Hazard ratios (HR) and 95% confidence intervals of all-cause mortality, myocardial infarction (MI), and stroke by the decile of weight change. We analyzed the HR for all-cause mortality, MI, and stroke in deciles of weight change, setting the stable weight group (7th decile; weight change range 0–1.5%) as the reference group. Adjusted for age, sex, alcohol drinking, smoking, regular exercise, income status, baseline fasting blood glucose, dyslipidemia, hypertension, waist circumferences, use of insulin and estimated glomerular filtration rate

Analysis according to decile groups of weight change revealed that the risk of all-cause mortality was lowest when there was no change in weight (seventh decile; weight change range 0–1.5%) and the risk of stroke increased significantly at the tenth decile of weight change (weight gain > 5.9%) (Fig. 2). When participants with end points that occurred within 1 year of follow-up were excluded, the HRs were slightly attenuated, but the overall U-shaped pattern of risk of all-cause mortality remained significant (Table 3). The results were nearly identical when data at diagnosis of DM was used instead of baseline data (data from the 2nd exam) in  model 3 of the Cox proportional hazards model (Additional file 1: Table S4). Additional file 1: Table S5 shows that in all 5 weight-change groups, the proportion of men, participants with hypertension and with insulin therapy were significantly higher among those who died compared to those who survived. Among the survivors there were a significantly higher proportion of participants performing regular exercise.

**Table 3:** Hazard ratios and 95% confidence intervals of all-cause mortality, myocardial infarction and stroke by weight-change categories; sensitivity analysis excluding subjects with the occurrence of end points within 1 year of follow-up

Weight change (%)	Myocardial infarction			Stroke			All-cause mortality		
	Events (n)	Incidence rate (per 1000 person-years)	HR (95% CI)	Events (n)	Incidence rate (per 1000 person-years)	HR (95% CI)	Events (n)	Incidence rate (per 1000 person-years)	HR (95% CI)
≥ − 10%	57	2.43	1.20 (0.91,1.58)	65	2.77	1.01 (0.78,1.30)	180	7.64	1.74 (1.48, 2.03)
− 10 ~ − 5%	197	2.27	1.13 (0.96,1.33)	197	2.27	0.90 (0.77,1.06)	433	4.97	1.22 (1.09, 1.36)
− 5 ~ 5%	870	2.09	1 (ref.)	1056	2.54	1 (ref.)	1614	3.87	1 (ref.)
5 ~ 10%	126	2.15	1.01 (0.83,1.22)	163	2.78	1.10 (0.94,1.30)	306	5.19	1.28 (1.14, 1.45)
≥ 10%	55	2.52	1.18 (0.89,1.55)	77	3.54	1.35 (1.06,1.70)	140	6.39	1.58 (1.33, 1.88)

Adjusted for age, sex, alcohol drinking, smoking, regular exercise, income status, baseline fasting glucose levels, dyslipidemia, hypertension, waist circumferences, insulin use and estimated glomerular filtration rate. *HR* hazard ratios, *CI* confidence intervals

### Subgroup analyses

We performed stratified analyses by age, sex, BMI category, and smoking status. We analyzed the HR for all-cause mortality in the weight-change groups, setting the stable weight group as the reference group after adjusting for all covariates.

**Fig. 3:**
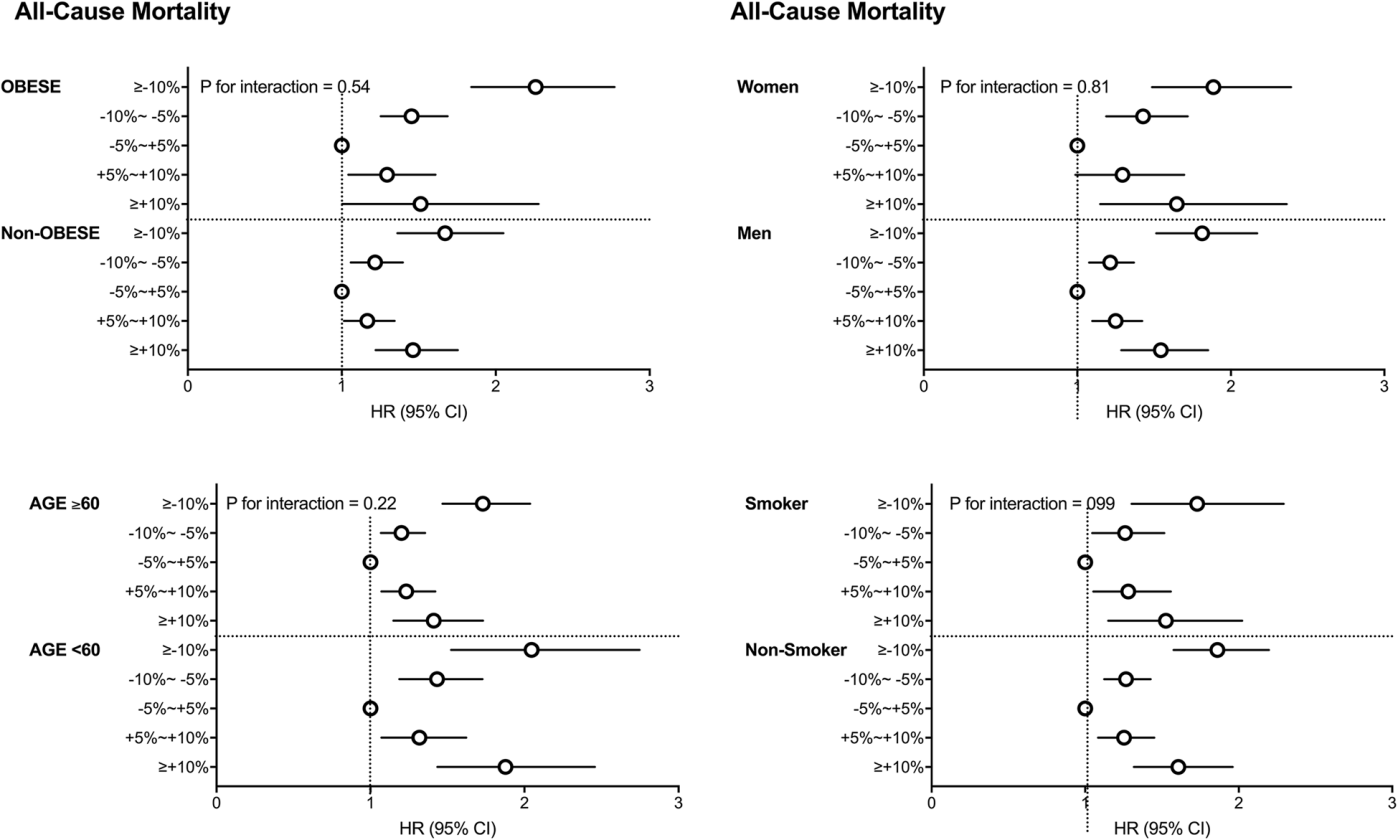
Subgroup analyses of association between the weight change categories and all-cause mortality stratified by obesity, age, sex and smoking category. Hazard ratios and 95% confidence intervals of myocardial infarction and stroke by the weight change categories. Adjusted for age, sex, alcohol drinking, smoking, regular exercise, income status, baseline fasting blood glucose, dyslipidemia, hypertension, waist circumferences,use of insulin and estimated glomerular filtration rate

**Fig. 4:**
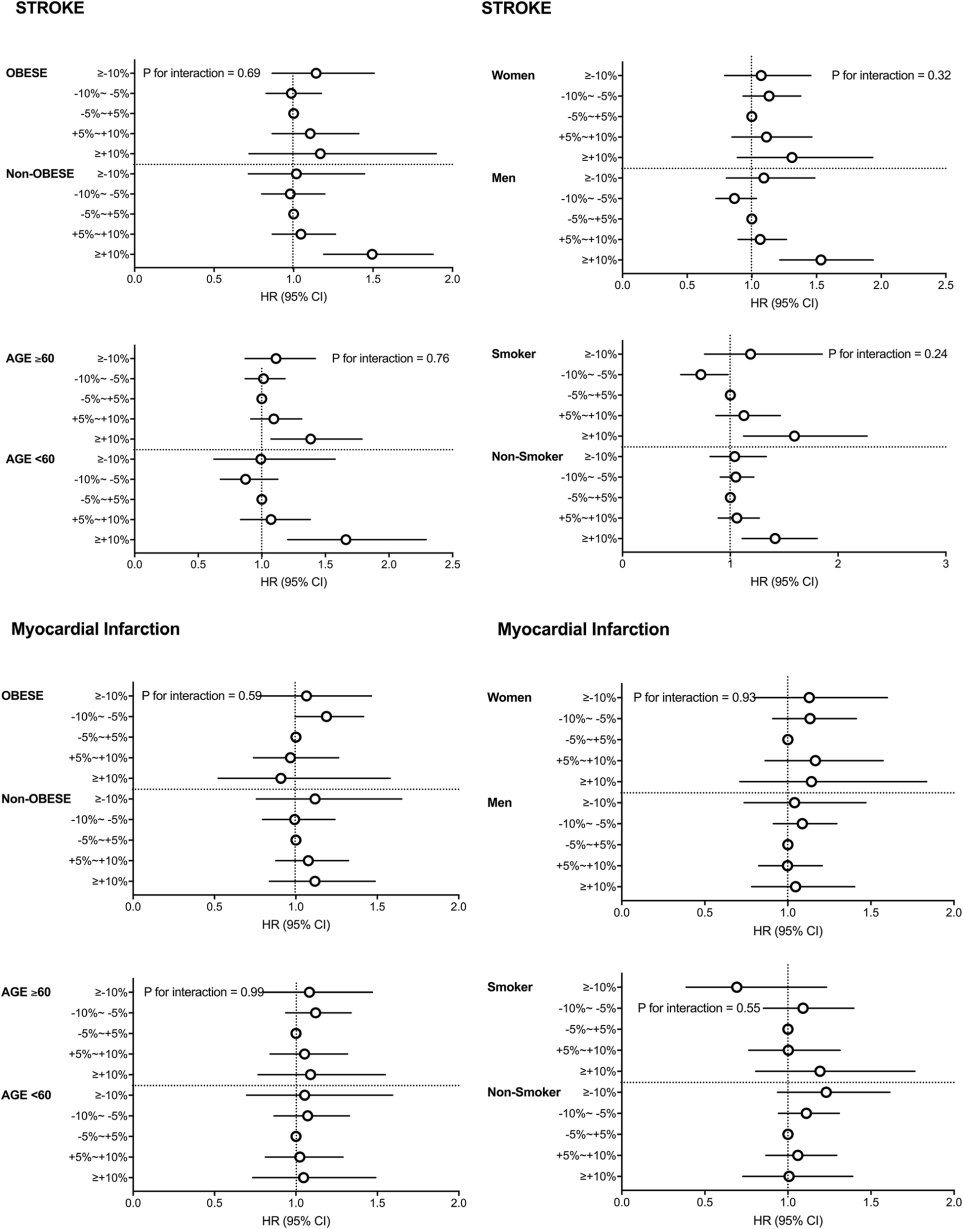
Subgroup analyses of association between the weight change categories and stroke, and myocardial infarction stratified by obesity, age, sex and smoking category. Hazard ratios and 95% confidence intervals of myocardial infarction and stroke by the weight change categories. Adjusted for age, sex, alcohol drinking, smoking, regular exercise, income status, baseline fasting blood glucose, dyslipidemia, hypertension, waist circumferences,use of insulin and estimated glomerular filtration rate

The risks of all-cause mortality showed a U-shaped curve according to weight change in all study subgroups (Fig. 3). Men who gained 10% weight had a higher HR for stroke (1.54, 95% CI 1.22–1.94), but women who gained 10% weight had no significant increase in risk for stroke compared with women with stable weight (HR 1.31, 95% CI 0.89–1.93) (Fig. 4). However, there was no interaction between weight change and sex, obesity, and age for the analysis of stroke (all P for interaction > 0.05).

## Discussion

Our data indicate that weight changes of more than 10% after diabetes diagnosis are more detrimental than a stable weight. We found that weight changes of more than 10% after diabetes diagnosis were associated with higher mortality and over 10% weight gain was associated with increased risk of stroke.

In our study, weight change after diagnosis of diabetes was not associated with the risk of MI. However, weight gain after a diagnosis of diabetes was related to an increased risk of stroke. Weight gain is a powerful risk factor for hypertension. It also adversely affects blood lipid profiles, increasing triglycerides, decreasing HDL cholesterol, and increasing oxidative stress. In this study, we found that weight gain ≥ 10% after a diagnosis of DM was associated with unfavorable metabolic profiles, including higher systolic and diastolic BP. Weight gain after a diagnosis of DM might reflect poor adherence to healthy lifestyle recommendations regarding diet and exercise for type 2 DM management [19]. We also found that weight gain ≥ 10% was related to an unhealthy lifestyle (current smoking/heavy alcohol drinking), as indicated in Table 1. It was previously reported that weight gain while taking metformin may be a marker for poor compliance with antidiabetic medication, which has been reported to be associated with higher mortality [20]. Weight gain may occur because of treatment with insulin or other hypoglycemic agents such as sulfonylurea. However, insulin-induced weight gain was not associated with adverse cardiovascular outcomes and mortality in patients with type 2 DM [21]. In our study, the association between weight changes and stroke and mortality did not change after adjustment for insulin use.

Decreased weight in the obese groups predicted a higher risk of all-cause mortality. Initially, it was hypothesized that a change in weight toward normal would be associated with a lower risk of all-cause mortality. However, weight gain or loss in patients with DM is associated with increased risk of all-cause mortality, regardless of their baseline BMI. Weight loss may be related to inappropriate dietary intake leading to a reduction in muscle mass rather than abdominal fat. The benefit of intentional weight loss could be confounded by unintentional weight loss because of the severity of diabetes [4]; i.e., weight loss may reflect poor control of diabetes, because higher blood glucose levels promote urinary glucose loss and subsequently, caloric loss [6]. However, in our study, the group with weight loss ≥ − 10% had relatively lower FBG both at the time of diagnosis of DM and 2 years later, compared with participants with a weight gain ≥ 10%. An intentional therapeutic weight loss was not associated with reduced mortality or cardiovascular morbidity in the subsequent 13 years in a cohort study of overweight patients with newly diagnosed type 2 DM [22]. The lowest mortality was seen among those patients who maintained their weight during the study period. Gregg et al. [23] demonstrated that the mere “intention to lose weight” in subjects both with and without DM was associated with reduced mortality regardless of whether they actually lost weight or not. In a previous study of weight history among participants in the Danish Diabetes Care in General Practice (DCGP) study [24], an average weight loss was seen after diagnosis in all age categories, which may suggest that weight loss is part of the natural history of diabetes. Indeed, 6 years after a diabetes diagnosis, patients with type 2 DM maintained an average weight loss of 2.5 kg. However, this weight change did not exceed 5%. Our study showed that regardless of weight gain or loss, ≥ 10% weight change is a risk factor for predicting all-cause mortality, and a weight gain of ≥ 10% is a risk factor for incident stroke.

Stroke is more frequently associated with diabetes in Asian than it is in patients with diabetes in Europe and North America [25]. The incidence of stroke was 1.5–2.5 times higher than that of MI in Japan. And shown in Table 2, the incidence of stroke was also higher than that of MI in Korea. The lack of association between weight gain and MI in our study may be related to the lower incidence of MI compared to stroke. Asian populations have excess visceral adiposity which may contribute to the overall metabolic risk even at lower levels of BMI [26]. In a Japanese study, minimal weight reduction (less than 3%) in the lifestyle intervention lowered the risk of developing DM by 53% over 3 years, similar to the risk reduction seen in the US Diabetes Prevention Program (58%) where the subjects lost 5–7% of body weight [27, 28]. Asian population was especially sensitive to adiposity [26]. Weight loss of 0.5–2.5 kg, especially centrally as shown by a decrease in waist circumference, when combined with some increase in physical activity, has beneficial effects on metabolic variables [27]. However, severe weight loss was associated with increased mortality in patients with DM, regardless of ethnicity [29]. In our study, we found that underweight and weight loss of more than 10% after a diabetes diagnosis were risk factors of all-cause mortality among patients with type 2 DM. The highest risk for mortality was among those underweight with a BMI < 18.5 kg/m^2^; while among the overweight and obese patients, risk for mortality was significantly lower than that of the normal weight. It was also reported that South Asians with normal weight at diagnosis of DM were more likely to die earlier by about 2.5 years compared to those who were obese at diagnosis [30]. The increased mortality in underweight and severe weight loss groups can be associated with various clinical factors, such as low body muscle mass, poor nutritional status, and occult illnesses [31]. Another explanation can have to do with the presence of “metabolically obese underweight” population that is in the underweight range on BMI but has an increased proportion of visceral fat and metabolic abnormalities, including dyslipidemia or insulin resistance [31]. Compared to normal BMI, there was an increased burden of coronary artery disease for BMI > 25 kg/m^2^ [32]. In that study, the hazard associated with BMI is mostly mediated by the presence of other metabolic risk factors. Subjects without metabolic syndrome had a lower hazard of adverse cardiovascular events according to BMI from 20 to 40 kg/m^2^ [32].

We also found that men who had a weight gain ≥ 10% had a higher risk for stroke compared with men with stable weight. But women who gained ≥ 10% weight had no significant increase in risk for stroke compared with women with stable weight. However, there was no interaction between weight change and sex for the analysis of stroke. The lack of association in women may be due to the high proportion of men (65%) participating in this study. It was reported that greater BMI increases during puberty was associated with risk of adult stroke in men [33, 34]. In that study, greater BMI increase during puberty was a strong predictor of an adult diagnosis of hypertension, suggesting that high blood pressure might be a mediating factor for the association between BMI increase through puberty and risk of adult stroke in men [33, 34].

Several prospective studies have reported that those who fluctuate in their body weight, with weight loss followed by weight gain or vice versa (weight cycling), have an increased risk of cardiovascular and all-cause mortality [35–37]. The effect of body weight fluctuation on incident DM depended on the presence of obesity at baseline [37]. The risk of DM decreased with an increase in the body weight fluctuation in subjects with a baseline BMI > 25 kg/m^2^. In that study, a greater reduction in body weight (weight loss) was observed with higher body weight fluctuation in obese subgroup [37]. In our study, we have examined the effect of weight changes, not cycling, on cardiovascular events and mortality. Moreover, the subjects of this study were all patients with new-onset type 2 DM.

The current study has several strengths. First, the most powerful strength of this study was that these data were based on a nationwide Korean population covering nearly 100% of Korean patients with type 2 DM. Second, we analyzed only patients with new-onset type 2 DM. The initial period after diagnosis of type 2 DM is critical for weight interventions to improve glycemia and risk factor control. Third, most studies of the effects of longitudinal weight changes have used questionnaires to identify self-reported body weight or past weight loss episodes and were not free from information bias. Our study used actual measurements of weight variables because these data were based on the physical examination during health checkups of the study participants.

We also acknowledge several limitations of our study. First, we did not know whether body weight changes were intentional or unintentional. Intentional weight loss in obese persons is associated with lower mortality. We cannot exclude that our results may be partly explained by residual confounding from pathological weight loss. In our study, all patients with prior or incident cancer were excluded. Moreover, sensitivity analysis excluding subjects with outcomes occurring in the first year of follow-up revealed similar results. Second, it is difficult to separate the effects of glycemic control, treatment, and weight change. Insulin is likely to be associated with poor glycemic control and weight loss. Insulin treatment tended to improve glycemia with weight gain. However, our results were unchanged after adjustment for use of insulin. Our study also limited the lack of data on diabetes treatments except insulin. Third, selection of study subjects who have received at least 2 health examinations might be a source of selection bias because men and employee subscribers were more likely to participate in the regular health check-up. Fourth, this was an observational study and therefore the association found between weight changes and end points may not be causal. Lastly, we did not know the causes of death: studies on causes of death according to weight change are required.

## Conclusion

Our study investigated the effect of weight change on the risk of mortality and CVD among patients with new-onset DM and suggested that weight changes ≥ 10% are generally detrimental than a stable weight. Although weight loss has been recommended for obese patients with type 2 DM, weight change might be detrimental especially in elderly persons [38]. Rather than weight loss, clinical interventions should target healthy lifestyle behaviors, particularly physical activity in obese adults. A study conducted in subjects with coronary heart disease showed that sustained physical activity, not weight loss, was associated with improved survival [39]. Recognizing the characteristics that discern survivors from non-survivors should be helpful to guide clinicians and public health practitioners in detecting at-risk individuals for early prevention [40]. We found that regardless of weight changes, there were a significantly higher proportion of participants performing regular exercise among the survivors.

## Additional file

**Additional file 1: Table S1.** Proportion of weight-change categories according to the body mass index (BMI) at diagnosis of diabetes mellitus. **Figure S1.** Kaplan-Meier estimates of cumulative incidence of all-cause mortality, myocardial infarction and stroke by the weight-changes for 2 years after diagnosis of diabetes mellitus. **Table S2.** Hazard ratios and 95% confidence intervals of Myocardial infarction and stroke by the weight-changes for 2 years after diagnosis of diabetes mellitus: analysis using sub-distribution hazards model, and mortality was considered as a competing risk. **Figure S2.** Association between weight change and the hazard ratios (HRs) (log scale) for myocardial infarction, stroke and all-cause mortality using restricted cubic splines. Restricted cubic spline curves were used to allow nonlinearity between weight change and outcomes. The X-axis represents body weight changes in %, while the Y-axis the risk of MI, stroke, and all-cause mortality. Shaded regions show 95% confidence limits. **Table S3.** Hazard ratios and 95% confidence intervals of Myocardial infarction, stroke and all-cause mortality according to baseline body mass index (BMI). **Table S4.** Hazard ratios and 95% confidence intervals of Myocardial infarction and stroke by the weight-changes for 2 years after diagnosis of diabetes mellitus: Sensitivity analysis adjusted with data at diagnosis of DM instead of baseline data. **Table S5.** Baseline characteristics that discern survivors from non-survivors in the different weight change groups.

## Abbreviations

CVD: cardiovascular disease
DM: diabetes mellitus
MI: myocardial infarctions
NHIS: National Health Insurance System
FBG: fasting blood glucose
BMI: body mass index
ICD: International Classification of Diseases
BP: blood pressure
