# Supplementary material for: Weight change and mortality and cardiovascular outcomes in patients with new-onset diabetes mellitus: a nationwide cohort study
Source: Cardiovasc Diabetol. 2019 Mar 19;18:36. doi: 10.1186/s12933-019-0838-9 (PMC6423842; doi:10.1186/s12933-019-0838-9)
Supplement: Supplementary file 1 — Additional file 1: Table S1. Proportion of weight-change categories according to the body mass index (BMI) at diagnosis of diabetes mellitus. Figure S1. Kaplan-Meier estimates of cumulative incidence of all-cause mortality, myocardial infarction and stroke by the weight-changes for 2 years after diagnosis of diabetes mellitus. Table S2. Hazard ratios and 95% confidence intervals of Myocardial infarction and stroke by the weight-changes for 2 years after diagnosis of diabetes mellitus: analysis using sub-distribution hazards model, and mortality was considered as a competing risk. Figure S2. Association between weight change and the hazard ratios (HRs) (log scale) for myocardial infarction, stroke and all-cause mortality using restricted cubic splines. Restricted cubic spline curves were used to allow nonlinearity between weight change and outcomes. The X-axis represents body weight changes in %, while the Y-axis the risk of MI, stroke, and all-cause mortality. Shaded regions show 95% confidence limits. Table S3. Hazard ratios and 95% confidence intervals of Myocardial infarction, stroke and all-cause mortality according to baseline body mass index (BMI). Table S4. Hazard ratios and 95% confidence intervals of Myocardial infarction and stroke by the weight-changes for 2 years after diagnosis of diabetes mellitus: Sensitivity analysis adjusted with data at diagnosis of DM instead of baseline data. Table S5. Baseline characteristics that discern survivors from non-survivors in the different weight change groups. [file 12933_2019_838_MOESM1_ESM.docx]

Table S1. Proportion of weight-change categories according to the body mass index (BMI) at diagnosis of diabetes mellitus

| Weight change (%) | BMI at diagnosis of diabetes mellitus | |
| --- | --- | --- |
|  | <25 kg/m^2^ | ≥ 25 kg/m^2^ |
| ≥ -10% | 2,496 (3.2%) | 4,398 (4.6%) |
| -10 ~ -5% | 10,123 (12.9%) | 15,323 (16.2%) |
| -5 ~ 5% | 51,721 (66.0%) | 66,898 (70.6%) |
| 5 ~ 10% | 9,578 (12.2%) | 6,544 (6.9%) |
| ≥ 10% | 4,437 (5.7%) | 1,611 (1.7%) |

Table S2. Hazard ratios and 95% confidence intervals of Myocardial infarction and stroke by the weight-changes for 2 years after diagnosis of diabetes mellitus: analysis using sub-distribution hazards model, and mortality was considered as a competing risk

| Weight change (%) | Events (n) | Incidence rate (per 1000 person-years) | Model 1 | Model 2 |
| --- | --- | --- | --- | --- |
| Myocardial infarction | | | | |
| ≥ -10% | 72 | 3.05 | 1.03 (0.82, 1.31) | 1.07 (0.84, 1.36) |
| -10 ~ -5% | 265 | 3.04 | 1.06 (0.93, 1.21) | 1.10 (0.96, 1.26) |
| -5 ~ 5% | 1188 | 2.84 | 1 (ref.) | 1 (ref.) |
| 5 ~ 10% | 174 | 2.95 | 1.05 (0.90, 1.23) | 1.03 (0.88, 1.21) |
| ≥ 10% | 68 | 3.09 | 1.10 (0.86, 1.41) | 1.06 (0.83, 1.36) |
| Stroke | | | | |
| ≥ -10% | 93 | 3.95 | 1.03 (0.83, 1.27) | 1.06 (0.85, 1.31) |
| -10 ~ -5% | 278 | 3.19 | 0.92 (0.81, 1.04) | 0.97 (0.85, 1.10) |
| -5 ~ 5% | 1375 | 3.29 | 1 (ref.) | 1 (ref.) |
| 5 ~ 10% | 207 | 3.51 | 1.10 (0.95, 1.27) | 1.08 (0.93, 1.25) |
| ≥ 10% | 107 | 4.89 | 1.49 (1.22, 1.82) | 1.45 (1.19, 1.77) |

Model 1: adjusted for age, sex, alcohol drinking, smoking, regular exercise and income status

Model 2: adjusted for model 1 plus baseline fasting glucose levels, dyslipidemia, hypertension, waist circumferences, use of insulin and estimated glomerular filtration rate

Table S3. Hazard ratios and 95% confidence intervals of Myocardial infarction, stroke and all-cause mortality according to baseline body mass index (BMI)

| BMI (kg/m^2^) | Events (n) | Incidence rate (per 1000 person-years) | Model 1 | Model 2 |
| --- | --- | --- | --- | --- |
| Myocardial infarction | |  |  |  |
| <18.5 | 28 | 5.94 | 1.61 (1.10, 2.37) | **1.66 (1.11, 2.49)** |
| 18.5-23 | 398 | 3.05 | 1 (ref.) | 1 (ref.) |
| 23-25 | 450 | 2.88 | 0.99 (0.87, 1.14) | 0.93 (0.80, 1.07) |
| 25-30 | 762 | 2.85 | 1.05 (0.93, 1.19) | 0.90 (0.77, 1.06) |
| ≥30 | 126 | 2.49 | 1.12 (0.92, 1.38) | 0.86 (0.65, 1.13) |
| Stroke |  |  |  |  |
| <18.5 | 33 | 7.02 | 1.37 (0.96, 1.95) | **1.44 (1.01, 2.08)** |
| 18.5-23 | 503 | 3.86 | 1 (ref.) | 1 (ref.) |
| 23-25 | 553 | 3.55 | 1.00 (0.89, 1.13) | 0.91 (0.80, 1.04) |
| 25-30 | 844 | 3.15 | 1.00 (0.89, 1.12) | **0.80 (0.70, 0.93)** |
| ≥30 | 127 | 2.51 | 1.00 (0.82, 1.22) | **0.67 (0.51, 0.87)** |
| All-cause mortality | |  |  |  |
| <18.5 | 105 | 22.06 | 1.98 (1.62, 2.42) | **1.93 (1.56, 2.38)** |
| 18.5-23 | 1050 | 7.99 | 1 (ref.) | 1 (ref.) |
| 23-25 | 768 | 4.89 | 0.67 (0.61, 0.73) | **0.66 (0.60, 0.73)** |
| 25-30 | 1051 | 3.90 | 0.60 (0.55, 0.65) | **0.58 (0.52, 0.65)** |
| ≥30 | 139 | 2.74 | 0.62 (0.52, 0.74) | **0.55 (0.44, 0.69)** |

Model 1: adjusted for age, sex, alcohol drinking, smoking, regular exercise and income status

Model 2: adjusted for model 1 plus baseline fasting glucose levels, dyslipidemia, hypertension, waist circumferences, use of insulin and estimated glomerular filtration rate

Table S4. Hazard ratios and 95% confidence intervals of Myocardial infarction and stroke by the weight-changes for 2 years after diagnosis of diabetes mellitus: Sensitivity analysis adjusted with data at diagnosis of DM instead of baseline data

| Weight change (%) | Events (n) | Incidence rate  (per 1000  Person-years) | Model 1 |
| --- | --- | --- | --- |
| Myocardial infarction | | | |
| ≥ -10% | 72 | 3.05 | 1.08 (0.85, 1.38) |
| -10 ~ -5% | 265 | 3.04 | 1.08 (0.94, 1.24) |
| -5 ~ 5% | 1188 | 2.84 | 1 (ref.) |
| 5 ~ 10% | 174 | 2.95 | 1.07 (0.91, 1.26) |
| ≥ 10% | 68 | 3.09 | 1.13 (0.87, 1.45) |
| Stroke |  |  |  |
| ≥ -10% | 93 | 3.95 | 1.09 (0.88, 1.35) |
| -10 ~ -5% | 278 | 3.19 | 0.95 (0.83, 1.08) |
| -5 ~ 5% | 1375 | 3.29 | 1 (ref.) |
| 5 ~ 10% | 207 | 3.51 | 1.04 (0.89, 1.20) |
| ≥ 10% | 107 | 4.89 | 1.31 (1.06, 1.61) |
| All-cause mortality | | | |
| ≥ -10% | 224 | 9.43 | 1.87 (1.62, 2.16) |
| -10 ~ -5% | 523 | 5.96 | 1.28 (1.16, 1.41) |
| -5 ~ 5% | 1862 | 4.43 | 1 (ref.) |
| 5 ~ 10% | 343 | 5.78 | 1.20 (1.06, 1.35) |
| ≥ 10% | 161 | 7.28 | 1.42 (1.20, 1.68) |

Model 1: adjusted for age, sex, alcohol drinking, smoking, regular exercise, income status, fasting glucose levels at diagnosis of DM, dyslipidemia at diagnosis of DM, hypertension at diagnosis of DM, waist circumferences at diagnosis of DM and use of insulin at diagnosis of DM

Table S5. Baseline characteristics that discern survivors from non-survivors in the different weight change groups

| Weight change (%) | ≥ -10% | | | -10~-5% | | | -5~+5% | | | 5~10% | | | ≥ 10% | | |
| --- | --- | --- | --- | --- | --- | --- | --- | --- | --- | --- | --- | --- | --- | --- | --- |
|  | Survivors | Non-survivors | P-value* | Survivors | Non-survivors | P-value* | Survivors | Non-survivors | P-value* | Survivors | Non-survivors | P-value* | Survivors | Non-survivors | P-value* |
| Age (years) | 56.8±12.0 | 68.3 ±11.4 | <.0001 | 56.5 ±10.8 | 66.5 ± 11.3 | <.0001 | 55.9±10.1 | 65.4± 10.4 | <.0001 | 54.7± 10.2 | 64.2 ± 10.6 | <.0001 | 53.9 ± 10.6 | 65.0± 12.3 | <.0001 |
| Sex (male) | 3182(48.0) | 137 (61.2) | 0.0001 | 14126(57.0) | 353 (67.5) | <.0001 | 77520 (66.8) | 1460 (78.5) | <.0001 | 11065(70.6) | 279 (81.3) | <.0001 | 4239(72.5) | 128(79.5) | 0.048 |
| BMI (kg/m^2^) | 23.1±3.1 | 21.5±3.1 | <.0001 | 24.4±3.0 | 23.3±3.2 | <.0001 | 25.7±3.2 | 24.5±3.2 | <.0001 | 26.3±3.4 | 25.0±3.5 | <.0001 | 26.6±3.6 | 25.0±3.3 | <.0001 |
| Height (cm) | 161.2±9.7 | 160.4±9.4 | 0.226 | 162.5± 9.3 | 160.7± 9.0 | <.0001 | 164.0±8.8 | 162.9± 8.4 | <.0001 | 164.5± 8.8 | 163.2 ± 8.4 | 0.006 | 164.8 ± 8.9 | 162.5 ± 8.8 | 0.0014 |
| Body weight (kg) | 60.2±11.4 | 55.5±10.0 | <.0001 | 64.6±11.3 | 60.2± 10.5 | <.0001 | 69.2±11.4 | 65.0± 10.6 | <.0001 | 71.33 ± 12.15 | 66.73 ± 11.54 | <.0001 | 72.4 ± 12.51 | 66.21 ± 10.67 | <.0001 |
| FBG (mg/dL) | 127.3±48.6 | 135.7±59.6 | 0.012 | 131.2 ±42 | 136.5±48.3 | 0.004 | 137.2±38.4 | 136.9±44.9 | 0.756 | 139.9 ± 38.6 | 138.9 ± 43.7 | 0.660 | 140.8 ± 42.0 | 135.4 ± 49.9 | 0.106 |
| Systolic BP (mmHg) | 123.5±14.8 | 125.9±17 | 0.019 | 125.3±14.4 | 128.5±15.9 | <.0001 | 127.6±14.3 | 130± 15.9 | <.0001 | 128.9 ± 14.3 | 131.8 ± 16.5 | <0.001 | 129.4 ± 14.9 | 130.9 ± 15.5 | 0.205 |
| Diastolic BP (mmHg) | 76.4 ± 9.8 | 75.9±9.7 | 0.539 | 77.5 ± 9.5 | 77.8±10.1 | 0.504 | 79.0 ±9.5 | 78.4± 10.2 | 0.003 | 79.8 ± 9.5 | 79.6± 10.2 | 0.827 | 80.2 ± 9.6 | 79.0 ± 10.2 | 0.114 |
| Hypertension (yes, %) | 3118 (47.0) | 134 (59.8) | <0.001 | 12128(49.0) | 339(64.9) | <.0001 | 63443(54.7) | 1185 (63.7) | <.0001 | 8972 (57.2) | 232 (67.8) | <.0001 | 3324 (56.9) | 116 (72.1) | <0.001 |
| Total cholesterol (mg/dL) | 184.1±40.8 | 182.9±43.0 | 0.666 | 188.8±40.6 | 188.1±44.0 | 0.671 | 191.2±40.1 | 185.9±41.1 | <.0001 | 188.6 ± 39.0 | 185.1 ± 45.8 | 0.105 | 188.2 ± 39.5 | 183.8 ± 42.6 | 0.171 |
| Dyslipidemia (yes, %) | 2945 (44.5) | 78 (34.8) | 0.004 | 11659(47.1) | 209(40.0) | 0.001 | 58848(50.7) | 689 (37.1) | <.0001 | 8143 (52.0) | 111(32.4) | <.0001 | 3069 (52.5) | 55 (34.2) | <.0001 |
| Waist circumferences (cm) | 80.4 ± 8.6 | 80.6±8.9 | 0.656 | 83.1 ± 8.1 | 83.2±8.0 | 0.745 | 86.5 ± 8.1 | 86.2±8.2 | 0.080 | 87.9 ± 8.4 | 87.5±8.9 | 0.331 | 88.6± 8.7 | 87.3±8.3 | 0.068 |
| Current smoker | 1407 (21.2) | 55 (24.6) | 0.232 | 6023 (24.3) | 147(28.2) | 0.041 | 31566(27.2) | 605(32.5) | <.0001 | 4560 (29.1) | 124(36.3) | 0.004 | 1763 (30.2) | 53 (32.9) | 0.453 |
| Heavy alcohol drinker | 326 (4.9) | 18 (8.1) | 0.035 | 1675 (6.8) | 52 (10.1) | 0.004 | 10612 (9.2) | 182 (9.9) | 0.321 | 1542 (9.9) | 43 (12.6) | 0.098 | 585 (10.1) | 22 (13.8) | 0.129 |
| Regular Exercise | 3536 (53.4) | 87 (38.84) | <.0001 | 13784(55.7) | 192(37.4) | <.0001 | 65836(56.8) | 821(44.3) | <.0001 | 8644 (55.3) | 164(48.1) | 0.009 | 3199 (54.8) | 65 (40.9) | 0.001 |
| Income (lower 25%) | 1423 (21.5) | 55 (24.6) | 0.266 | 4797 (19.4) | 135(25.8) | <0.001 | 22213(19.1) | 448(24.1) | <.0001 | 3262 (20.8) | 89 (25.9) | 0.020 | 1254 (21.4) | 36 (22.4) | 0.777 |
| Use of Insulin (yes) | 158 (2.4) | 11 (4.9) | 0.016 | 319 (1.3) | 20 (3.8) | <.0001 | 1242 (1.07) | 49 (2.6) | <.0001 | 331 (2.1) | 12 (3.5) | 0.079 | 249 (4.3) | 14 (8.7) | 0.007 |

Data are expressed as the means ± SD, or n (%). *P-values are measured using t-test (continuous variables) and Chi-square test (categorical variables) comparing baseline characteristics in survivors versus non-survivors in the different weight change groups. BMI, body mass index; FBG, fasting blood glucose; BP, blood pressure

Figure S1. Kaplan-Meier estimates of cumulative incidence of all-cause mortality, myocardial infarction and stroke by the weight-changes for 2 years after diagnosis of diabetes mellitus

0

1

2

3

4

5

6

7

**0**

**2**

**4**

**6**

**8**

**Myocardial Infarction**

**Time(Years)**

**Incidence probability (%)**

~ -10%

-10% ~ -5%

-5% ~ 5%

+5% ~ 10%

+10% ~

0

1

2

3

4

5

6

7

**0**

**1**

**2**

**3**

**4**

**STROKE**

**Time(Years)**

**Incidence probability (%)**

~-10%

-10% ~-5%

-5%~+5%

+5%~+10%

+10%~

0

1

2

3

4

5

6

7

**0**

**2**

**4**

**6**

**8**

**All-Cause Mortality**

**Time(Years)**

**Incidence probability (%)**

~ -10%

**-**10% ~ -5%

**-**5% ~ 5%

+5% ~ 10%

+10% ~

Supplemental Figure S2. Association between weight change and the hazard ratios (HRs) (log scale) for myocardial infarction, stroke and all-cause mortality using restricted cubic splines. Restricted cubic spline curves were used to allow nonlinearity between weight change and outcomes. The X-axis represents body weight changes in %, while the Y-axis the risk of MI, stroke, and all-cause mortality. Shaded regions show 95% confidence limits.

-1.0

-0.5

0.0

0.5

1.0

1.5

**Smooth Hazard Ratio for Weight change (Myocardial infarction)**

Weight change (%) (%)

log HR(95% CI)

-50

-40

-30

-20

-10

0

10

20

30

40

50

Ref = 5.47945

-0.5

0.0

0.5

1.0

1.5

**Smooth Hazard Ratio for Weight change (Stroke)**

Weight change (%)

log HR(95% CI)

-50

-40

-30

-20

-10

0

10

20

30

40

50

Ref = -5.15464

0.0

0.5

1.0

1.5

2.0

**Smooth Hazard Ratio for Weight change (All-cause mortality)**

Weight change (%)

log HR(95% CI)

-50

-40

-30

-20

-10

0

10

20

30

40

50

Ref = 0.82645
